# Endocrine determinants of MASLD: mechanisms and therapeutic targets

**DOI:** 10.3389/fendo.2026.1967494

**Published:** 2026-10-05

**Authors:** Richa Chaturvedi, Anu Mathew, Aprajita Pradhan, Monika Goyal, Monika Sharma, Varsha Kachroo, Chhavi Agrawal, Aishwarya Krishnamurthy, Surabhi Atreja, Ashish Atreja

**Affiliations:** 1 Advance Centre for Obesity, Diabetes and Endocrinology (ACODE), Indraprastha Apollo Hospital, Delhi, India; 2 Department of Endocrinology, Fortis Hospital, Gurugram, Haryana, India; 3 Department of Endocrinology, Max Superspecialty Hospital, Dwarka, Delhi, India; 4 Department of Endocrinology, Yatharth Super Speciality Hospital, New Delhi, India; 5 Department of Endocrinology, Aakash Hospital, Dwarka, Delhi, India; 6 Department of Endocrinology, Fortis Escorts Heart Institute, Delhi, India; 7 Department of Endocrinology, Max Super Speciality Hospital, Ghaziabad, Uttar Pradesh, India; 8 Division of Cardiovascular Medicine, UC Davis Medical Center, Sacramento, CA, United States; 9 Division of Gastroenterology and Hepatology, Stanford University School of Medicine, Stanford, Redwood, CA, United States

**Keywords:** adipokines, GH/IGF-1, glucocorticoids, gut-liver axis, hypogonadism, incretins, insulin resistance, MASH

## Abstract

Metabolic dysfunction-associated steatotic liver disease (MASLD) affects approximately 30% of adults worldwide, yet is still framed largely as a hepatological disorder despite being driven substantially by endocrine and metabolic dysfunction. This review proposes a unified endocrine framework organised around seven hormonal axes, presenting each in terms of molecular pathogenesis, clinical presentation, and therapeutic implications. Insulin resistance is the central driver, coupling chronic caloric excess to hepatic *de novo* lipogenesis through selective hepatic insulin resistance, although genetic variants in *PNPLA3* and *TM6SF2* can uncouple steatosis from metabolic risk. Adipose tissue dysfunction contributes through adipocyte senescence, adipokine imbalance, and inflammatory adipose–liver signalling. Reduced hepatic thyroid hormone sensitivity, even in euthyroid individuals, predicts steatosis and fibrosis and underpins the first approved liver-directed therapy. Gonadal steroids exert sexually dimorphic effects, with testosterone deficiency, menopausal oestrogen loss, and polycystic ovary syndrome representing distinct risk states. Glucocorticoid dysregulation is stage-dependent, shifting from enhanced hepatic cortisol clearance in steatosis to local amplification in steatohepatitis. The gut-liver-endocrine axis, encompassing incretin, bile acid, and fibroblast growth factor signalling, has proved the most therapeutically tractable. Reduced GH/IGF-1 signalling, whether from pituitary disease or obesity-induced functional suppression, promotes both steatosis and fibrogenesis. Biological sex and life-course influences spanning developmental programming and ageing further modify these determinants. Two agents, resmetirom and semaglutide, now have US FDA accelerated approval for noncirrhotic MASH with moderate-to-advanced fibrosis, supporting the principle that correcting axis-specific endocrine dysfunction can produce measurable hepatic benefit; however, both approvals are accelerated and await confirmatory outcome data, while others remain investigational. An endocrine framework offers both a coherent account of pathogenesis and a rational basis for biomarker-guided, phenotype-targeted therapy.

## 1 Introduction

Metabolic dysfunction–associated steatotic liver disease (MASLD), the term adopted by international consensus in 2023 to replace non-alcoholic fatty liver disease (NAFLD), affects approximately 30% of adults worldwide and is the most prevalent chronic liver disorder (1, 2). Its rise parallels the global increase in obesity and type 2 diabetes mellitus (T2DM), with which it shares substantial mechanistic overlap. The disease spans a histological spectrum from simple steatosis to metabolic dysfunction associated steatohepatitis (MASH), fibrosis, and cirrhosis, with hepatocellular carcinoma (HCC) and cardiovascular disease as its principal causes of mortality (3). Despite this well-characterised natural history, the endocrine drivers that shape an individual patient’s trajectory remain incompletely integrated into clinical practice.

At its core, MASLD begins with a mismatch between energy intake and metabolic capacity. Chronic caloric excess overwhelms the storage and oxidative capacity of adipose tissue and the liver, promoting ectopic lipid accumulation and the resulting insulin resistance. Insulin resistance (IR), a state in which target tissues respond inadequately to insulin, is well established as the central defect (4, 5). In the liver specifically, this takes the form of selective (or partial) hepatic insulin resistance: the liver fails to suppress glucose production in response to insulin, yet paradoxically retains, and even amplifies, insulin’s capacity to drive fat synthesis. This selective defect explains why hyperglycaemia and hepatic fat accumulation so often coexist. GH/IGF-1axis, adipose tissue dysfunction, disordered adipokine secretion, and gut microbiota-driven inflammation are recognised as further contributors, and European Association for the Study of the Liver (EASL), American Association for the Study of Liver Diseases (AASLD) and American Association of Clinical Endocrinology (AACE) guidelines now incorporate metabolic risk assessment into MASLD algorithms (3, 6–8).

The endocrine determinants of MASLD, however, extend well beyond the insulin-adipose axis to include thyroid hormone sensitivity, growth hormone/IGF-1 axis, gonadal steroid action, and hypothalamic-pituitary-adrenal (HPA) dysregulation. The arrival of endocrine-targeted therapies - the thyroid hormone receptor-β agonist resmetirom and the GLP-1 receptor agonist semaglutide, both granted accelerated approval by the US FDA for noncirrhotic MASH with fibrosis stages F2-F3-makes an integrated framework both timely and clinically urgent, as the rational use of these agents depends on the axis-specific biology they target.

The endocrine contributions to MASLD have been reviewed previously, most comprehensively by Hutchison and colleagues, who examined the disease in relation to non-diabetic endocrinopathies (9). The present review differs in three respects. First, rather than setting insulin resistance aside, we position it as the central axis through which several other hormonal disturbances exert their hepatic effects. Second, we advance a convergence framework in which mechanistically distinct axes act on a shared set of intrahepatic processes, and we examine how biological sex and life-course factors modify all of them. Third, the therapeutic landscape has since changed substantially: resmetirom and semaglutide have received regulatory approval for MASH, and phase 2 and 3 data have emerged for incretin, FGF21, and FXR-directed agents, enabling a treatment discussion structured explicitly by stage of evidence.

This review proposes a unified endocrine framework for MASLD, organised around seven hormonal axes: insulin resistance and glucose metabolism; adipose tissue and adipokines; and the thyroid, GH/IGF-1, gonadal, HPA, and gut-liver endocrine axes. For each axis, we synthesise the molecular pathogenesis, clinical presentation, and therapeutic implications. We then consider two cross-cutting modifiers of these axes - biological sex and the life-course influences of early-life exposure and ageing - before summarising the emerging endocrine-targeted therapeutic landscape. Our aim is a mechanistically grounded, cross-speciality synthesis that supports earlier identification of at-risk patients, more precise endocrine phenotyping for therapeutic stratification, and a rational basis for future research.

## 2 Major endocrine axes in MASLD

The liver occupies a unique position in metabolic physiology as the first organ to receive absorbed nutrients through the portal circulation and the principal site at which systemic fuel availability is sensed and buffered. Across the fed–fasted cycle, hepatocytes alternate between net lipogenesis and glycogen storage after feeding and glycogenolysis, gluconeogenesis and fatty acid oxidation during fasting - transitions that are directed almost entirely by hormonal signals rather than by substrate availability alone. This work is not uniformly distributed: metabolic zonation across the hepatic lobule assigns oxidative metabolism and gluconeogenesis preferentially to periportal hepatocytes and lipogenesis and glycolysis to the pericentral zone, which is also where steatosis characteristically begins. Hepatic lipid handling reflects the balance of four fluxes: uptake of circulating fatty acids, *de novo* synthesis, oxidation, and export as very-low-density lipoprotein, each under separate hormonal control. Steatosis represents the failure of this balance rather than a single defective step, and the endocrine axes considered below act by shifting different components of it. Although presented separately for clarity, these axes are neither independent nor equally weighted; each is engaged through distinct upstream mechanisms, yet all converge on a common set of intrahepatic processes that culminate in stellate cell activation and fibrogenesis, as summarised in **Figure 1**.

**Figure 1:**
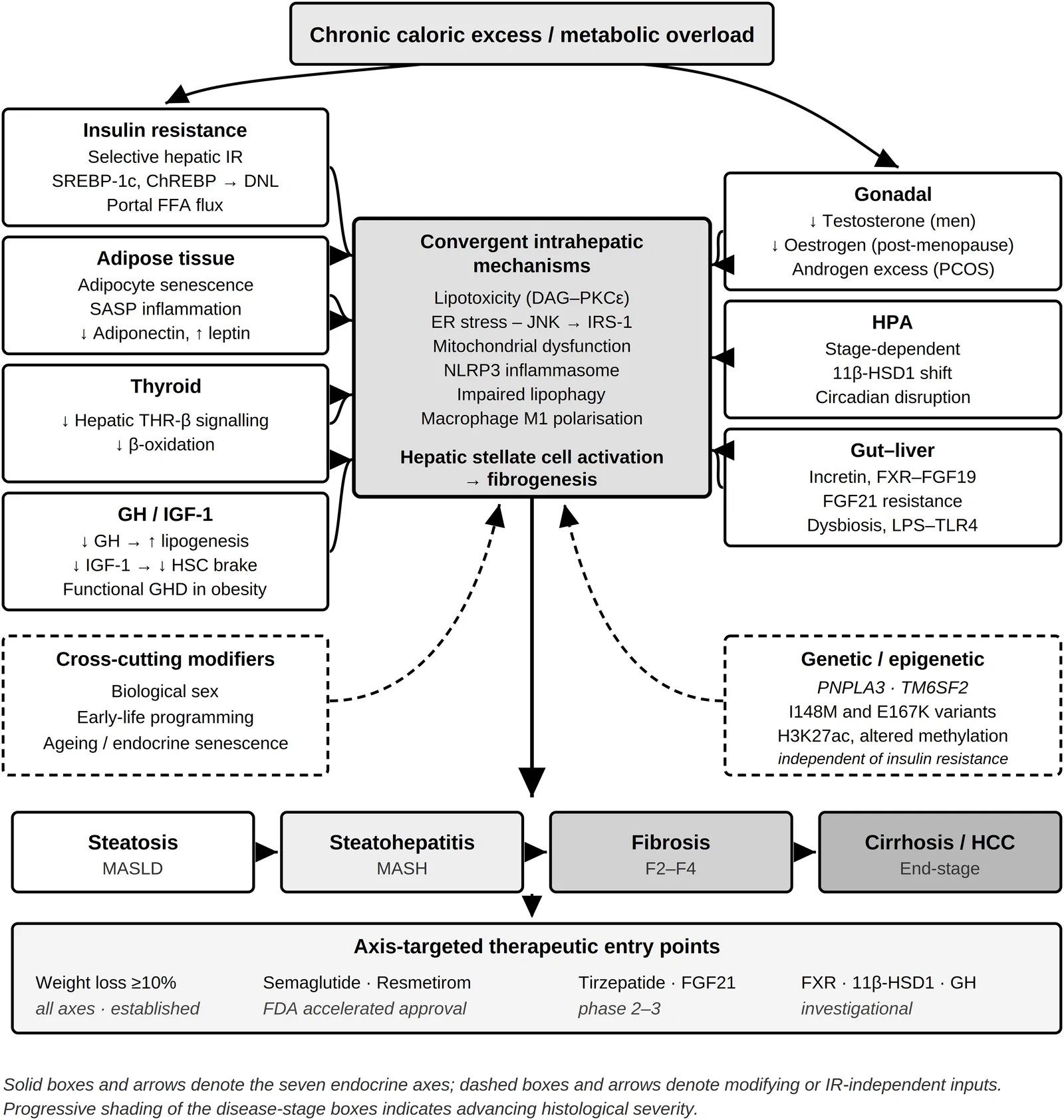
Integrated endocrine framework for MASLD.

Chronic caloric excess destabilises seven endocrine axes: insulin resistance and glucose metabolism; adipose tissue and adipokines; and the thyroid, GH/IGF-1, gonadal, hypothalamic-pituitary-adrenal (HPA), and gut-liver-endocrine axes (solid boxes and arrows). Although each axis is engaged through distinct upstream mechanisms, they converge on a shared set of intrahepatic processes: lipotoxic diacylglycerol–protein kinase C-epsilon (DAG–PKCϵ) signalling, endoplasmic reticulum stress with c-Jun N-terminal kinase (JNK)-mediated degradation of insulin receptor substrate-1 (IRS-1), mitochondrial dysfunction, NLRP3 inflammasome activation, impaired lipophagy, and macrophage M1 polarisation, which together drive hepatic stellate cell activation and fibrogenesis. Two further inputs are shown with dashed borders. Biological sex, early-life developmental programming and ageing act as cross-cutting modifiers of every axis and contribute to the disease’s phenotypic heterogeneity. Genetic and epigenetic susceptibility, including the *PNPLA3* I148M and *TM6SF2* E167K variants and durable chromatin modifications at lipogenic loci, constitutes a parallel route to hepatic lipid accumulation that acts independently of insulin resistance and can produce steatosis and fibrosis in metabolically near-normal individuals. Progressive shading of the disease-stage boxes indicates advancing histological severity. The lower panel indicates the principal therapeutic entry points at each axis, distinguishing established and approved interventions from those still under investigation.

### 2.1 Insulin resistance and glucose metabolism

#### 2.1.1 Molecular pathogenesis

Insulin normally coordinates fuel handling across the liver and adipose tissue. In adipose tissue, it suppresses lipolysis, restraining the release of free fatty acids (FFAs); in the liver, it simultaneously suppresses glucose production and promotes lipid synthesis. In the fed state, these actions keep circulating glucose and fatty acids within a narrow range. Insulin resistance disrupts this coordination, and in the liver, it does so selectively (4, 5).

The process begins with chronic caloric excess. When adipose tissue can no longer buffer surplus energy, insulin loses its ability to restrain lipolysis, and a sustained stream of FFAs is delivered to the liver. At the same time, the liver develops selective (partial) hepatic insulin resistance: it no longer responds to insulin’s suppression of gluconeogenesis, yet remains responsive to insulin’s stimulation of lipogenesis via sterol regulatory element-binding protein-1c (SREBP-1c). This selectivity produces the defining paradox of MASLD- unrestrained hepatic glucose output and unrestrained fat synthesis occurring simultaneously, which is why hyperglycaemia and hepatic steatosis so frequently coexist (10, 11). Portal FFA delivery and SREBP-1c- driven *de novo* lipogenesis (DNL) together supply the substrate for hepatic triglyceride accumulation; DNL alone contributes up to 30% of the intrahepatic triglyceride pool in MASLD, compared with less than 5% in healthy individuals (5). Lipogenesis is not driven by insulin alone. Carbohydrate-responsive element-binding protein (ChREBP) is activated directly by glucose and fructose metabolites independently of insulin signalling, providing a second, substrate-sensing route to *de novo* lipogenesis. This explains why dietary sugar, particularly fructose, which bypasses the principal regulatory step of glycolysis, promotes hepatic lipogenesis even when insulin signalling is impaired, and why the two arms are additive in the insulin-resistant liver (10).

Once established, this lipogenic state becomes self-reinforcing, and the individual pathways are best understood as interlocking loops. The accumulating lipids are not inert. Lipotoxic diacylglycerols (DAGs) activate protein kinase C-epsilon (PKCϵ), which phosphorylates the insulin receptor at threonine-1160, directly blunting insulin signalling. Thus, the stored fat deepens the very resistance that generated it (12). In parallel, the surplus of saturated lipids overwhelms the endoplasmic reticulum (ER); the resulting ER stress activates c-Jun N-terminal kinase (JNK), which degrades insulin receptor substrate-1 (IRS-1) and further impairs signal transduction (13, 14). Superimposed on both, chronic nutrient surplus persistently activates mammalian target of rapamycin complex 1 and its effector S6 kinase 1 (mTORC1/S6K1), which simultaneously amplify SREBP-1c transcription and phosphorylate IRS-1 to sustain the resistant state (13). Because these three loops converge on the same defect, relieving the hepatic lipid burden interrupts all of them at once, explaining why even modest reductions in liver fat markedly restore insulin sensitivity (12).

Genetics can uncouple this sequence. Loss-of-function variants in the gene *PNPLA3* (I148M), which encodes patatin-like phospholipase domain-containing protein 3, a lipase that remodels lipid droplets in hepatocytes, and *TM6SF2* (E167K), which encodes transmembrane 6 superfamily member 2, a protein required for hepatic very-low-density lipoprotein (VLDL) export, both cause fat to accumulate in the liver independently of insulin resistance. Carriers may therefore develop steatosis and a high risk of fibrosis despite a lean profile, with practical implications for screening (15). This predisposition is reinforced epigenetically: histone H3 lysine 27 acetylation (H3K27ac) at lipogenic gene promoters and altered methylation at the *PNPLA3/TM6SF2* loci create a durable “metabolic memory” that sustains DNL even after weight loss (16).

#### 2.1.2 Clinical presentation

Clinically, the relationship between MASLD and type 2 diabetes mellitus (T2DM) is bidirectional and quantifiable: 55-70% of patients with T2DM have MASLD, and MASLD increases the risk of incident T2DM two- to threefold (5). Beyond co-occurrence, T2DM independently predicts advanced fibrosis, cirrhosis, and HCC, independent of steatosis and body mass index (BMI). This excess risk reflects the downstream consequences of hyperinsulinaemia and hyperglycaemia: activation of hepatic stellate cells, accumulation of advanced glycation end products (AGEs), and oxidative stress-mediated hepatocellular injury. Because hepatic insulin resistance also undermines systemic glycaemic control, MASLD and T2DM are most usefully assessed and managed as part of a single cardiometabolic syndrome (4, 8).

#### 2.1.3 Therapeutic implications

The self-reinforcing nature of hepatic lipotoxicity explains why interventions that reduce the substrate load are so effective. Sustained weight loss of ≥10% through lifestyle modification remains the most reliable way to reduce hepatic fat and, with it, insulin resistance, and can achieve MASH resolution and fibrosis regression in a substantial proportion of patients (17). Among pharmacotherapies targeting this axis, the glucagon-like peptide-1 (GLP-1) receptor agonist semaglutide improves insulin sensitivity and hepatic steatosis and, following the phase 3 ESSENCE trial, received US FDA accelerated approval in 2025 for noncirrhotic MASH with moderate-to-advanced fibrosis (18) while the dual GLP-1/glucose-dependent insulinotropic polypeptide (GIP) receptor agonist tirzepatide achieved MASH resolution without worsening of fibrosis in up to 73% of participants in the phase 2 SYNERGY-NASH trial (19). A phase 3 clinical-outcomes trial is ongoing, and tirzepatide is not yet approved for MASH. The glucagon/GLP-1 dual agonist survodutide improved MASH without worsening of fibrosis in a phase 2 trial and has received FDA Breakthrough Therapy designation for noncirrhotic MASH (20). Sodium-glucose cotransporter-2 (SGLT2) inhibitors reduce hepatic fat content in patients with T2DM and confer additional cardiometabolic benefit (21).

### 2.2 Adipose tissue and adipokines

#### 2.2.1 Molecular pathogenesis

Adipose tissue is an endocrine organ, and its dysfunction during chronic caloric excess is a primary driver of MASLD (22). The causal sequence begins with the same energy surplus that underlies hepatic insulin resistance. As visceral adipose tissue is forced to store more lipid than it can safely accommodate, adipocytes become hypertrophic and locally hypoxic, stabilising hypoxia-inducible factor-1α (HIF-1α) and eventually entering a state of cellular senescence (23). Crucially, insulin resistance and adipose dysfunction reinforce one another in a feedback loop: insulin resistance impairs the normal suppression of lipolysis, driving further lipid accumulation within adipose tissue; the resulting senescent adipocytes adopt a senescence-associated secretory phenotype (SASP), sustaining the release of interleukin-6 (IL-6), interleukin-1β (IL-1β), and tumour necrosis factor-α (TNF-α) (24, 25). This secretory output polarises resident macrophages towards the pro-inflammatory M1 phenotype and propagates low-grade systemic inflammation, which in turn deepens insulin resistance. The consequence for the liver is twofold: impaired insulin suppression of lipolysis floods the portal circulation with free fatty acids (FFAs), fuelling hepatic lipotoxicity and *de novo* lipogenesis, while systemic inflammation promotes hepatic stellate cell activation and fibrogenesis. Adipose dysfunction is therefore best understood as an upstream determinant that both initiates and sustains the full MASLD spectrum (26).

Superimposed on this inflammatory shift is a disturbance in adipokine balance. Adiponectin, which acts through AdipoR1/R2 receptors and AMP-activated protein kinase (AMPK)-peroxisome proliferator-activated receptor-α (PPARα) signalling to suppress DNL, promote fatty acid β-oxidation, and limit NLRP3 inflammasome activation, declines as visceral fat expands. Its decline independently predicts progression from steatosis to MASH and fibrosis (27). Conversely, leptin rises, but functional leptin resistance blunts its central effects while its peripheral actions persist: hepatic stellate cells are activated through JAK/STAT3 and transforming growth factor-β1 (TGF-β1) signalling, accelerating fibrosis independently of steatosis severity (28). Beyond this classical pairing, a wider network of adipokines, including asprosin (which stimulates hepatic glucose production via hypothalamic AgRP neurons), adipsin, chemerin, and retinol-binding protein-4 (RBP4), is increasingly implicated, though their individual contributions to MASLD remain under investigation (6, 29). Adipose tissue also communicates with the liver via extracellular vesicles (EVs) carrying microRNA cargo: miR-155 suppresses hepatocyte PPARγ and promotes M1 polarisation, whereas miR-34a targets sirtuin-1 (SIRT1), thereby impairing PPARα-mediated fatty acid oxidation. Circulating EV-microRNA profiles are being explored as non-invasive MASH biomarkers but remain unvalidated for clinical use (30).

The role of adipose tissue *browning* is more tentative and warrants cautious interpretation. White adipose tissue can acquire thermogenic, energy-dissipating “beige” characteristics, mediated by peroxisome proliferator-activated receptor-γ coactivator 1-α (PGC-1α) and uncoupling protein 1 (UCP1). In obesity and MASLD this browning capacity appears diminished, plausibly reducing the thermogenic disposal of surplus lipid and favouring its ectopic deposition in the liver. However, the evidence linking impaired browning directly to human MASLD is largely preclinical and associative, and this pathway should be regarded as mechanistically plausible but not yet clinically established (31).

#### 2.2.2 Clinical presentation

The clinical signature of adipose dysfunction in MASLD is the distribution of fat rather than its total amount. Visceral adiposity, reflecting the portal FFA flux described above, correlates more closely with hepatic steatosis and fibrosis than BMI. Accordingly, the visceral-to-subcutaneous adipose tissue ratio (VAT/SAT), quantified by MRI or CT, outperforms BMI as a predictor of steatosis severity and MASH progression and correlates with intrahepatic triglyceride content and liver stiffness. This has practical consequences: patients with predominantly visceral or ectopic fat deposition, including those who are lean by BMI, may carry disproportionate hepatic risk, and body composition rather than weight alone is the more informative clinical measure.

#### 2.2.3 Therapeutic implications

Because adipose dysfunction is upstream, restoring adipose health is central to durable MASLD resolution, and current and emerging therapies act at several points in the sequence. Sustained weight loss reduces adipocyte stress and portal FFA flux and remains foundational. Among incretin-based agents, the GLP-1 receptor agonist semaglutide (FDA-approved for MASH with fibrosis) and the dual GLP-1/GIP receptor agonist tirzepatide (approved for T2DM and obesity, and investigational in MASH) reduce visceral fat and adipose inflammation (19, 32). Agents targeting the specific mechanisms above are at earlier stages: senolytics (the dasatinib-plus-quercetin combination and navitoclax) aim to clear SASP-bearing senescent adipocytes and have shown reductions in adipose inflammation in early-phase trials (33); β3-adrenergic receptor agonists (mirabegron) are being explored to restore thermogenic adipose activity, though, consistent with the tentative browning evidence above, their hepatic benefit in MASLD remains unproven (34). The thyroid hormone receptor-β agonist resmetirom also promotes hepatocyte fatty acid oxidation and is discussed in full under the thyroid axis. The key distinction here is between established weight-directed therapy and still-investigational agents targeting senescence and thermogenesis.

### 2.3 Thyroid axis

#### 2.3.1 Molecular pathogenesis

Thyroid hormones are central regulators of hepatic lipid and energy metabolism, and disturbances in thyroid signalling have emerged as a distinct endocrine determinant of MASLD. The liver is a principal target of thyroid hormone action, mediated predominantly by the thyroid hormone receptor β (THR-β) isoform. Triiodothyronine (T3), the active hormone, promotes hepatic fatty acid β-oxidation and mitochondrial biogenesis while restraining *de novo* lipogenesis (DNL). It is also essential for mitophagy - the selective autophagic clearance of damaged mitochondria that maintains oxidative capacity (35, 36). Through these actions, T3 favours lipid clearance over lipid storage, and any reduction in hepatic thyroid hormone signalling shifts this balance towards fat accumulation.

Two distinct disturbances are relevant to MASLD. The first is overt or subclinical hypothyroidism, in which reduced circulating thyroid hormone directly lowers hepatic β-oxidation and mitochondrial activity, promoting steatosis. The second, increasingly recognised, is a reduction in thyroid hormone action despite normal circulating hormone concentrations, a state termed euthyroid, meaning biochemically normal thyroid function. It is useful to distinguish three levels at which this may occur. At the central level, altered sensitivity of the pituitary-thyroid feedback loop produces a mismatch between thyroid-stimulating hormone and free thyroxine (FT4) at a given hormone concentration. At the peripheral level, the deiodinase enzymes that govern the conversion of FT4 to the active triiodothyronine (T3) may be up- or down-regulated, altering systemic T3 availability. At the hepatic tissue level, thyroid hormone action may be reduced through decreased intracellular T3 availability, altered local deiodinase activity, or diminished THR-β responsiveness, so-called intrahepatic hypothyroidism. These levels are related but not equivalent, and the last is most directly implicated in MASLD pathogenesis and is the target of liver-selective THR-β agonism (35, 37).

#### 2.3.2 Clinical presentation

Hypothyroidism is associated with increased prevalence and severity of MASLD, and this association persists across the spectrum from steatosis to fibrosis (38, 39). More striking is the evidence from euthyroid populations: even within the normal reference range, markers of reduced thyroid hormone sensitivity are linked to greater hepatic steatosis and higher fibrosis risk. Impaired central sensitivity to thyroid hormone, quantified by indices such as the Thyroid Feedback Quantile-based Index (TFQI), and a higher free T3 to free T4 (FT3/FT4) ratio have each been associated with greater liver fibrosis and higher controlled attenuation parameter (CAP) values, a measure of steatosis, in euthyroid adults with overweight or obesity (40, 41). These two indices, however, interrogate different levels of the axis and should not be interpreted interchangeably. TFQI reflects the set-point of central pituitary–thyroid feedback, whereas the FT3/FT4 ratio is a marker of peripheral deiodinase-mediated conversion. This distinction resolves an apparent paradox: an elevated FT3/FT4 ratio indicates *increased* peripheral generation of active hormone, which in early metabolic dysfunction most plausibly represents a compensatory response to rising adiposity and energy surplus rather than a state of thyroid hormone deficiency. Its association with hepatic steatosis therefore likely marks the underlying metabolic disturbance rather than reduced hepatic thyroid hormone action per se.

Neither index measures intrahepatic thyroid hormone signalling directly, and no validated circulating biomarker of hepatic THR-β activity currently exists, a significant gap, given that this is the level at which resmetirom acts. Both TFQI and the FT3/FT4 ratio are therefore best regarded as promising research tools for identifying a thyroid-related MASLD phenotype rather than as validated instruments for clinical risk stratification or treatment selection (40, 41).

#### 2.3.3 Therapeutic implications

The recognition that hepatic thyroid hormone signalling can be selectively targeted has led to the first liver-directed endocrine therapy for MASH. Resmetirom is an orally active, liver-selective THR-β agonist. Its design exploits two forms of selectivity: it is preferentially taken up by the liver, and it acts on the β rather than the α isoform of the receptor, thereby stimulating the beneficial hepatic β-oxidation and lipid-clearance effects of thyroid hormone while largely sparing the cardiac and skeletal effects mediated by THR-α. In the phase 3 MAESTRO-NASH trial, resmetirom achieved both MASH resolution and fibrosis improvement relative to placebo, leading to its regulatory approval, the first specifically for MASH with fibrosis (42). This establishes proof of concept that correcting reduced hepatic thyroid hormone action, rather than circulating hormone levels, is therapeutically effective. Whether patients identified as having impaired thyroid hormone sensitivity by indices such as TFQI derive disproportionate benefit is a logical and important hypothesis, but one that remains to be tested in prospective, biomarker-stratified trials.

### 2.4 Growth hormone/IGF-1 axis

#### 2.4.1 Molecular pathogenesis

Growth hormone (GH) and its principal mediator insulin-like growth factor-1 (IGF-1) regulate hepatic lipid handling through actions that are partly distinct from one another. GH acts directly on hepatocytes via the GH receptor–STAT5 pathway to suppress *de novo* lipogenesis and promote fatty acid oxidation and triglyceride export; liver-specific deletion of the GH receptor in mice produces hepatic steatosis, insulin resistance and raised circulating free fatty acids; restoring hepatic GHR normalised free fatty acid levels whereas IGF-I infusion did not, indicating that this is a direct hepatic GH effect rather than one mediated through IGF-I (43). IGF-1, produced largely by the liver in response to GH, has a different and complementary target: it acts on hepatic stellate cells to inhibit their activation and induce a p53-dependent senescent, non-fibrogenic state, thereby functioning as an endogenous antifibrotic signal (44). Loss of GH signalling therefore promotes steatosis, while loss of IGF-1 signalling removes a brake on fibrogenesis.

The relevance of this axis extends well beyond frank pituitary disease. Obesity and insulin resistance suppress GH secretion, reducing pulse amplitude and total daily output, and hepatic GH resistance further blunts IGF-1 generation. The result is a state of functional, acquired GH deficiency in which circulating GH is low, and IGF-1 is low-normal despite an intact pituitary. This links the GH/IGF-1 axis directly to the adipose and insulin-resistance axes discussed above and places it among the mechanisms by which caloric excess is translated into hepatic injury.

GH secretion is itself sexually dimorphic, with consequences for the liver. In rodents, GH is secreted in a markedly pulsatile pattern in males and a more continuous pattern in females; because hepatic STAT5 responds differently to pulsatile versus continuous receptor stimulation, this pattern difference drives extensive sex-specific hepatic gene expression, including genes governing lipid handling (45). A comparable but less pronounced sex difference exists in humans. Oestrogen further modulates the axis by inducing suppressors of cytokine signalling, which inhibit GH receptor-JAK2-STAT5 signalling and produce a degree of hepatic GH resistance; this underlies the reduction in circulating IGF-1 seen with oral but not transdermal oestrogen administration (46). The GH/IGF-1 axis therefore represents one mechanistic route by which gonadal steroid status is translated into sexually dimorphic hepatic phenotypes.

#### 2.4.2 Clinical presentation

Adult GH deficiency (AGHD) is associated with a substantially increased prevalence of MASLD and with more advanced histology, including steatohepatitis and fibrosis, and a significant proportion of patients with hypopituitarism develop MASLD in the absence of obesity (47). Conversely, in the general population with overweight or obesity, lower serum IGF-1 correlates with greater intrahepatic lipid content and, in patients with biopsy-proven disease, with fibrosis stage (48). AGHD should therefore be considered in patients with MASLD and a history of pituitary disease, cranial irradiation, or traumatic brain injury, and hypopituitarism is a recognised cause of severe MASLD in lean individuals. Routine IGF-1 measurement in unselected MASLD is not currently indicated, since low-normal values are common in obesity and do not reliably distinguish acquired functional suppression from organic deficiency.

#### 2.4.3 Therapeutic implications

In patients with organic AGHD, GH replacement reduces hepatic fat and improves histological features of steatohepatitis, providing direct evidence that restoring this axis is hepatoprotective (47). Whether GH-axis augmentation benefits the much larger population with functional suppression is under investigation. In a randomised, placebo-controlled trial in HIV-associated MASLD, the GHRH analogue tesamorelin reduced hepatic fat fraction measured by magnetic resonance spectroscopy and limited histological fibrosis progression over 12 months, though the population was specific and long-term histological effects remain undetermined (49). In a randomised, placebo-controlled trial in adults with overweight or obesity and MASLD, enrolment restricted to those with serum IGF-1 at or below the age-specific third quartile, six months of low-dose GH administration reduced intrahepatic lipid and improved MRI-derived measures of hepatic inflammation and fibrosis, without worsening glycaemic measures (50). No GH-axis agent is approved for MASLD, and the strategy remains investigational; key uncertainties include the durability of benefit, effects on insulin sensitivity, and the appropriate patient population.

### 2.5 Gonadal axis

#### 2.5.1 Molecular pathogenesis

Gonadal steroids exert direct, sexually distinct effects on hepatic lipid metabolism, and their dysregulation contributes to MASLD in ways that differ fundamentally between men and women. In men, testosterone restrains hepatic fat accumulation by promoting lipid oxidation, improving insulin sensitivity, and limiting visceral adipose expansion. Testosterone deficiency reverses these effects, increasing visceral adiposity and the portal free fatty acid flux that drives hepatic steatosis, while lowering insulin sensitivity, thereby establishing a self-reinforcing cycle in which hypogonadism and metabolic dysfunction aggravate one another (51–53). In women, oestrogen is broadly hepatoprotective, acting through oestrogen receptor-α to suppress *de novo* lipogenesis, support mitochondrial fatty acid oxidation, and restrain hepatic inflammation and stellate-cell activation. 17β-estradiol additionally modulates the NLRP3 inflammasome, contributing to its anti-inflammatory hepatic effect (54). The loss of oestrogen at menopause removes this protection and shifts adipose distribution towards a visceral pattern, accelerating hepatic lipid accumulation (55).

Polycystic ovary syndrome (PCOS) is a distinct, premenopausal route to MASLD, driven not by oestrogen deficiency but by androgen excess. Hyperandrogenism in PCOS promotes visceral fat storage and is closely linked to insulin resistance and compensatory hyperinsulinaemia, which together increase hepatic DNL and the risk of steatosis, independently of body weight (56, 57). PCOS therefore illustrates that androgen deficiency in men and androgen excess in women can converge on the same hepatic outcome through shared downstream effects on visceral adiposity and insulin resistance.

#### 2.5.2 Clinical presentation

These mechanisms produce recognisable clinical patterns. In men, hypogonadism is linked to a higher prevalence and severity of MASLD, and low testosterone frequently coexists with the metabolic syndrome and type 2 diabetes. The relationship is bidirectional, as hepatic and visceral fat accumulation suppresses testosterone (52, 53). In women, MASLD prevalence rises markedly after menopause, narrowing and eventually reversing the premenopausal sex difference in disease burden (55). PCOS is independently associated with MASLD across weight categories: meta-analytic data show a substantially increased prevalence of hepatic steatosis in women with PCOS compared with age- and BMI-matched controls, identifying PCOS as an important and often under-recognised risk marker warranting hepatic assessment (57, 58). A dedicated section below considers the broader, cross-cutting patterns of sexual dimorphism reflected in these observations.

#### 2.5.3 Therapeutic implications

Management of the gonadal contribution to MASLD is currently centred on correcting the underlying hormonal disturbance where a clear indication exists, rather than on gonadal-targeted therapy for MASLD itself. In men with established hypogonadism, testosterone replacement improves insulin sensitivity, reduces visceral and hepatic fat, and ameliorates features of the metabolic syndrome, as shown in observational and registry data. However, testosterone is not an approved treatment for MASLD, and randomised trial evidence for hepatic endpoints remains limited (52). In women with PCOS, management follows established PCOS guidelines, including weight management, insulin sensitisation, and, where appropriate, agents that reduce androgen excess; GLP-1 receptor agonists are increasingly used for their combined weight and metabolic benefits (58). Menopausal hormone therapy has a plausible mechanistic rationale for mitigating post-menopausal hepatic risk but is not indicated for MASLD, and its use is governed by broader menopausal and cardiovascular considerations. Across all three contexts, the clinically actionable step is recognition, namely screening hypogonadal men, post-menopausal women, and women with PCOS for hepatic steatosis and fibrosis, while gonadal-hormone-directed therapy for MASLD itself remains investigational.

### 2.6 Hypothalamic-pituitary-adrenal axis

#### 2.6.1 Molecular pathogenesis

Glucocorticoids are potent regulators of hepatic lipid and glucose metabolism, and both systemic and tissue-level disturbances have been implicated in MASLD. Cortisol acting on the hepatic glucocorticoid receptor (GR) promotes gluconeogenesis, drives visceral fat redistribution, and favours intrahepatic lipid accumulation, a profile closely resembling MASLD. Overt cortisol excess, as in Cushing’s syndrome, causes florid steatosis but is rare; the more common disturbance involves altered regulation of glucocorticoid availability within the liver itself (59, 60).

Intrahepatic glucocorticoid exposure reflects the balance between local regeneration by 11β-hydroxysteroid dehydrogenase type 1 (11β-HSD1), which converts inactive cortisone to cortisol, and clearance by A-ring reductases. Human studies across the disease spectrum show that these arms shift in opposite directions with disease stage. In simple steatosis, hepatic 11β-HSD1 activity is reduced, and 5α-reductase activity is increased, so hepatic cortisol clearance predominates, with a compensatory rise in adrenal glucocorticoid production. In steatohepatitis, this reverses: 11β-HSD1 expression and activity increase, localising to periseptal hepatocytes and CD68-positive macrophages within inflamed septa, while 5α-reductase falls and GRα rises, together maximising intrahepatic glucocorticoid exposure (61). Consistent with impaired clearance in advanced disease, hepatic expression of *AKR1D1*, which encodes steroid 5β-reductase, declines as steatosis, inflammation and fibrosis worsen (62). Rather than a uniform tissue-specific glucocorticoid excess, the axis is therefore dysregulated in a stage-dependent manner, with both systemic and local mechanisms contributing.

A proposed downstream consequence of sustained GR activation is impaired lipophagy, the selective autophagic degradation of hepatocyte lipid droplets, which would limit triglyceride mobilisation and favour progression to steatohepatitis. This derives largely from experimental models and awaits confirmation in human disease (63). Circadian disruption of the normal diurnal cortisol rhythm has also been linked experimentally to disordered hepatic lipid handling, though human evidence remains associative (64).

#### 2.6.2 Clinical presentation

The HPA contribution to MASLD is usually subtle. Cushing’s syndrome is an uncommon but recognised cause of severe steatosis and warrants consideration alongside other cushingoid features. Far more often, the disturbance is subclinical: mild autonomous cortisol secretion and functional HPA activation associated with chronic stress and the metabolic syndrome are linked to visceral adiposity and hepatic steatosis (59). Importantly, the raised cortisol production seen in early steatosis is compensatory for increased hepatic clearance rather than primary adrenal pathology, which may explain why routine testing is typically unremarkable (61). There is no established indication for routine HPA assessment in unselected MASLD.

#### 2.6.3 Therapeutic implications

Therapeutic interest centres on inhibiting local cortisol regeneration rather than lowering systemic cortisol, and trial results align with the stage-dependent biology. In a randomised phase 2 study, the selective 11β-HSD1 inhibitor AZD4017 produced no significant overall reduction in hepatic fat, but did reduce liver fat in the subgroup with both NASH and type 2 diabetes, the group in which hepatic 11β-HSD1 activity is expected to be elevated (65). This suggests that the class’s modest overall efficacy may reflect biological heterogeneity rather than a failed target, supporting a stratified approach.

An important caveat follows from the cell-type specificity of glucocorticoid action in the liver. Cortisol does not act solely on hepatocytes: hepatic stellate cells also express the glucocorticoid receptor (GR), and GR signalling in these cells functions as an endogenous antifibrotic brake, suppressing stellate cell proliferation and limiting their transdifferentiation into collagen-secreting myofibroblasts, thereby restraining the pro-fibrogenic transcriptional programme that drives matrix deposition. Intrahepatic cortisol availability therefore has opposing consequences in the two compartments, promoting lipid accumulation in hepatocytes while simultaneously suppressing fibrogenesis in stellate cells. The picture is further complicated by the fact that glucocorticoid action is not uniformly antifibrotic across hepatic cell types: GR signalling restrains fibrogenesis in stellate cells but appears to promote it in hepatic macrophages, so the net effect of reducing intrahepatic cortisol depends on the relative contribution of each compartment (66).

This creates a genuine therapeutic tension. By reducing the regeneration of active cortisol within the liver, 11β-HSD1 inhibition lowers the glucocorticoid signal available to all three cell types: the intended effect is relief of the hepatocyte lipid burden, but an unintended consequence may be release of the GR-mediated brake on stellate cell activation. Consistent with this, murine studies have shown that 11β-HSD1 deficiency or pharmacological inhibition exacerbates myofibroblast activation and hepatic fibrogenesis (67). Steatosis and fibrosis may thus respond to this intervention in opposite directions.

The clinical implication is specific: trials of 11β-HSD1 inhibitors in MASLD cannot be evaluated on hepatic fat content alone, since an apparently favourable steatosis result could coexist with fibrosis progression. Histological or validated non-invasive fibrosis endpoints are essential. No 11β-HSD1 inhibitor is approved for MASLD, and the strategy remains experimental. Addressing upstream HPA and circadian drivers- sleep, stress, and management of the metabolic syndrome- is mechanistically rational but unproven for hepatic outcomes.

### 2.7 Gut-liver-endocrine axis

#### 2.7.1 Molecular pathogenesis

The gut communicates with the liver through an integrated endocrine and microbial network, which has become one of the most therapeutically productive areas in MASLD. Three hormonal systems and one microbial system operate within this axis, and each is considered below.

The first is incretin signalling. Glucagon-like peptide-1 (GLP-1) and glucose-dependent insulinotropic polypeptide (GIP) are gut-derived hormones released after eating that enhance insulin secretion, promote satiety, and slow gastric emptying. Although the liver expresses few GLP-1 receptors, incretin action reduces hepatic fat indirectly by improving systemic insulin sensitivity, lowering body weight, and reducing the substrate load reaching the liver (19, 68).

The second is bile acid signalling via the farnesoid X receptor (FXR). Bile acids activate FXR in the intestine and liver. FXR activation in the intestine induces fibroblast growth factor 19 (FGF19), which travels to the liver to suppress bile acid synthesis and modulate hepatic lipid and glucose metabolism. FXR activation in the liver directly restrains *de novo* lipogenesis and gluconeogenesis. Disruption of FXR–FGF19 signalling contributes to the dysregulated lipid handling observed in MASLD and provides a rationale for FXR-directed therapy (69, 70).

The third is the hepatokine fibroblast growth factor 21 (FGF21). Produced mainly by the liver, FGF21 acts on adipose tissue and the central nervous system to increase fatty acid oxidation, improve insulin sensitivity, and promote energy expenditure. MASLD is associated with elevated FGF21 levels and a state of FGF21 resistance. Pharmacological FGF21 analogues are designed to overcome this resistance and restore its beneficial metabolic effects (71, 72).

The fourth component is the gut microbiota, and the evidence here must be interpreted with particular care. In MASLD, the intestinal microbiome exhibits altered composition (dysbiosis) and reduced barrier integrity, allowing microbial products such as lipopolysaccharide (LPS) to enter the portal circulation and activate hepatic Toll-like receptor 4 (TLR4) signalling, thereby promoting inflammation and fibrogenesis (73). Microbial metabolism of bile acids and production of short-chain fatty acids further link the microbiome to the FXR and incretin systems. However, it is important to distinguish correlation from causation: much of the human evidence shows that specific microbial signatures are associated with MASLD and its severity, rather than establishing that these signatures cause it (7). Causal inference rests largely on animal models, including faecal microbiota transplantation and germ-free colonisation experiments, which show that a MASLD-associated microbiome can transmit aspects of the phenotype, but the extent to which these findings translate to human disease remains incompletely defined (73, 74). The microbiota is therefore best regarded as a genuine and mechanistically plausible contributor, whose precise causal weight in human MASLD is still being established.

#### 2.7.2 Clinical presentation

Unlike the preceding axes, the gut–liver–endocrine axis has not yet yielded a discrete clinical phenotype or a validated diagnostic marker. Its clinical relevance is currently indirect and lies chiefly in its exceptional therapeutic tractability. Circulating FGF21, bile acid profiles, and microbiome-derived signatures are under investigation as biomarkers of disease activity or treatment response, but none is yet established for routine clinical use.

#### 2.7.3 Therapeutic implications

This axis has produced the most active pipeline in MASLD therapeutics, spanning approved and investigational agents. Incretin-based therapy is the most advanced: the GLP-1 receptor agonist semaglutide holds US FDA accelerated approval for noncirrhotic MASH with F2–F3 fibrosis on the basis of the phase 3 ESSENCE trial (18), and the dual GLP-1/GIP receptor agonist tirzepatide has demonstrated MASH resolution in the phase 2 SYNERGY-NASH trial (19), with a phase 3 outcomes trial (SYNERGY-Outcomes) under way. Both act largely by reducing weight and improving systemic insulin sensitivity (19, 32). FGF21 analogues (efruxifermin, pegozafermin) have shown reductions in hepatic fat and improvements in fibrosis in phase 2b-3 trials, representing a mechanistically distinct, still-investigational approach (72, 75). FXR-directed therapy has had a more mixed trajectory: the steroidal FXR agonist obeticholic acid improved fibrosis in trials but was limited by pruritus and adverse lipid effects (76), prompting the development of non-steroidal FXR agonists and engineered FGF19 analogues (aldafermin), now in trials, none yet approved (69, 70, 77). Along this axis, the therapeutic principle is that modulating gut-derived endocrine signals can confer hepatic benefit without directly acting on the liver. Except for approved incretin agents, these strategies remain investigational.

## 3 Sexual dimorphism in MASLD

The endocrine determinants of MASLD do not operate the same way in men and women. Biological sex shapes the disease’s prevalence, histological pattern, progression rate, and hormonal drivers. Across most of adult life, MASLD is more prevalent and tends to be more severe in men than in age-matched premenopausal women (78). This protection wanes after menopause, when declining oestrogen and a shift towards visceral fat distribution raise MASLD prevalence in women to meet or exceed that in men of the same age (79). Premenopausal androgen excess, as in polycystic ovary syndrome, with an associated insulin-resistant state overrides the usual female protection and independently elevates hepatic risk, irrespective of weight (80).

The sex difference in MASLD is best understood not as a fixed attribute but as a reproductive life-course trajectory. Earlier age at menarche is associated with higher MASLD prevalence in large cross-sectional data, although this relationship is partly mediated by adult adiposity (81), and reproductive exposures in adulthood further modulate risk in both directions: a history of gestational diabetes substantially increases subsequent MASLD prevalence (82), whereas longer duration of breastfeeding is protective in parous women (83). The timing of menopause matters as well, with earlier menopause conferring higher risk in long-term cohort data (84). These observations align with the recently proposed reproductive resilience hypothesis, which holds that female reproductive signalling networks, extending beyond classical sex steroids, are integral to systemic metabolic maintenance, and that their decline sets the tempo of sex-specific ageing across organs, including the liver (85).

Importantly, sexual dimorphism extends beyond the gonadal axis and modulates other endocrine determinants of MASLD. Sex differences in body fat distribution, with men accumulating visceral and ectopic fat more readily and premenopausal women favouring subcutaneous storage, mean that the adipose-tissue and insulin-resistance pathways described earlier are engaged to different degrees and at different thresholds in each sex (86). Thyroid hormone signalling, thyroid autoimmunity, and reference ranges for thyroid function differ between men and women, plausibly influencing the thyroid-related MASLD phenotype (38). The GH/IGF-1 axis is similarly dimorphic: sex differences in GH secretory pattern, amplified by oestrogen-induced hepatic GH resistance, produce sexually divergent hepatic gene expression and represent a mechanistic bridge between gonadal steroid status and the liver (87). Glucocorticoid biology, HPA-axis reactivity, and the tissue activity of 11β-hydroxysteroid dehydrogenase type 1 also show sex-related differences that may translate into differential hepatic glucocorticoid exposure (88). Even within the gut–liver axis, gut microbiota composition and circulating bile acid and incretin profiles differ by sex, adding another layer of dimorphism to disease susceptibility and, potentially, treatment response (89).

These differences have practical implications that are only beginning to be addressed (90). Much of the foundational MASLD evidence base, including in preclinical models, has historically been generated in male animals and male-predominant cohorts, which risks obscuring female-specific mechanisms and may limit the generalisability of both risk models and therapeutic data.

A sex-informed approach, accounting for menopausal status, recognising PCOS and hypogonadism as sex-specific risk states, and analysing therapeutic responses by sex, is likely to improve both risk stratification and trial design (90).

## 4 Life course modifiers of endocrine axes

Early-life exposures and developmental programming. The developmental origins of health and disease concept holds that the intrauterine and early postnatal environment durably shapes metabolic and endocrine set-points. Maternal obesity, gestational diabetes, and both undernutrition and overnutrition during pregnancy are each associated with an increased risk of offspring metabolic dysfunction and hepatic steatosis in later life (91). The proposed mechanisms map directly onto the axes discussed above: An adverse maternal metabolic environment programmes offspring insulin sensitivity and adipose tissue development, alters hypothalamic–pituitary-adrenal axis reactivity and glucocorticoid signalling, and influences the establishment of the neonatal gut microbiota, with effects on the gut-liver axis that may persist into adulthood. Epigenetic mechanisms, including DNA methylation and histone modifications at metabolic gene loci, provide a plausible molecular substrate for this programming, offering a route by which an early-life exposure can produce a durable “metabolic memory”. Early postnatal factors, such as infant feeding and rapid early weight gain, further modify this trajectory (92). The practical implication is that MASLD risk is, in part, established well before adulthood, and that maternal and early-life metabolic health represent potential windows for primordial prevention.

Ageing and endocrine senescence. At the opposite end of the life-course, ageing remodels the same endocrine axes in ways that favour MASLD. Ageing is accompanied by a progressive decline in the function of multiple hormonal systems: insulin sensitivity falls, growth hormone and gonadal steroid secretion decline-the latter representing, in women, the loss of the reproductive signalling networks discussed above (85), and thyroid hormone action and HPA-axis regulation shift with age. In parallel, body composition changes, with characteristic increases in visceral and ectopic fat and decreases in skeletal muscle mass, and this sarcopenic, visceral-adipose phenotype, along with cellular senescence, amplifies the adipose-tissue and insulin-resistance pathways central to MASLD (93, 94).

Together, early-life programming and age-related endocrine remodelling frame MASLD as a disease whose endocrine determinants operate across the entire lifespan. This perspective identifies distinct at each end, with primordial and primary prevention directed at maternal and early-life metabolic health, and, in older adults, attention to the combined burden of sarcopenia, visceral adiposity, and multi-axis endocrine decline.

## 5 Endocrine targeted therapeutics: an integrated view

The therapeutic implications of each endocrine axis have been considered in the relevant sections above. Taken together, they reveal a field rapidly transitioning from a single foundation in weight loss to a diversified, mechanism-based armamentarium, and several insights emerge (**Table 1**).

**Table 1:** Endocrine-targeted therapies in MASLD, by target axis and stage of evidence.

Agent (class)	Mechanism of action	Target axis	Stage of evidence	Status in MASLD
Lifestyle weight loss ≥10%	Reduces substrate load, adipose stress and insulin resistance across multiple axes	All axes	RCT/prospective cohort (17)	Established first-line; backbone of all management
Resmetirom (THR-β agonist)	Liver-selective thyroid hormone receptor-β agonism; ↑ hepatic β-oxidation, ↓ *de novo* lipogenesis	Thyroid	Phase 3 RCT (MAESTRO-NASH) (42)	US FDA accelerated approval (2024): noncirrhotic MASH, F2–F3
Semaglutide (GLP-1 RA)	Incretin receptor agonism; weight loss and improved systemic insulin sensitivity	Gut–liver/incretin	Phase 3 RCT (ESSENCE) (18, 32)	US FDA accelerated approval (2025): noncirrhotic MASH, F2–F3
Tirzepatide (dual GLP-1/GIP RA)	Dual incretin receptor agonism; greater weight reduction and metabolic improvement	Gut–liver/incretin	Phase 2 RCT (SYNERGY-NASH); phase 3 outcomes trial ongoing (19)	Approved for T2DM and obesity; not approved for MASH
Survodutide (dual GLP-1/glucagon RA)	Glucagon receptor co-agonism adds direct hepatic lipid-lowering to incretin effect	Gut–liver/incretin	Phase 2 RCT; phase 3 (LIVERAGE) ongoing (20)	Not approved; FDA Breakthrough Therapy designation
SGLT2 inhibitors	Glycosuria-mediated calorie loss; reduced hepatic fat and cardiometabolic risk	Insulin resistance/glucose	Phase 3/RCT (T2DM populations) (21)	Approved for T2DM; not approved for MASLD
Efruxifermin (FGF21 analogue)	Long-acting FGF21 receptor agonism; ↑ fatty acid oxidation, ↑ insulin sensitivity	Gut–liver/hepatokine	Phase 2b RCT completed; phase 3 ongoing (72)	Investigational
Pegozafermin (FGF21 analogue)	Glycopegylated FGF21 analogue; hepatic fat and fibrosis reduction	Gut–liver/hepatokine	Phase 2b RCT (ENLIVEN) completed; phase 3 ongoing (75)	Investigational
Obeticholic acid (steroidal FXR agonist)	FXR agonism; ↓ bile acid synthesis, ↓ lipogenesis	Gut–liver/bile acid	Phase 3 RCT (76)	Not approved for MASH; limited by pruritus and adverse lipid effects
Non-steroidal FXR agonists	FXR agonism with improved tolerability profile	Gut–liver/bile acid	Phase 2 (70)	Investigational
Aldafermin (engineered FGF19 analogue)	FGF19 pathway agonism; ↓ bile acid synthesis, ↓ hepatic fat	Gut–liver/bile acid	Phase 2 (77)	Investigational
Tesamorelin (GHRH analogue); GH replacement	Restoration of GH/IGF-1 signalling; ↓ hepatic *de novo* lipogenesis, ↑ lipid oxidation	GH/IGF-1	Phase 2 RCT (HIV-associated MASLD); RCT in adult GH deficiency (47, 49, 50)	GH approved for adult GH deficiency; not approved for MASLD
11β-HSD1 inhibitors (e.g. AZD4017)	Blockade of intrahepatic cortisone-to-cortisol regeneration	HPA	Phase 2 RCT (mixed; benefit limited to MASH + T2DM subgroup) (65)	Experimental; fibrosis endpoints required
Senolytics (dasatinib + quercetin; navitoclax)	Clearance of senescent adipocytes and the senescence-associated secretory phenotype	Adipose	Early-phase/preclinical (33)	Experimental
β3-adrenergic agonists (mirabegron)	Stimulation of adipose thermogenesis/browning	Adipose	Exploratory/preclinical (34)	Experimental; hepatic benefit unproven
Testosterone replacement	Restoration of androgen signalling; ↓ visceral fat, ↑ insulin sensitivity	Gonadal	Observational/limited RCT (52, 53)	Indicated for diagnosed hypogonadism only; not approved for MASLD

FGF, fibroblast growth factor; FXR, farnesoid X receptor; GH, growth hormone; GHRH, growth hormone-releasing hormone; GIP, glucose-dependent insulinotropic polypeptide; GLP-1, glucagon-like peptide-1; 11β-HSD1, 11β-hydroxysteroid dehydrogenase type 1; HPA, hypothalamic–pituitary–adrenal; IGF-1, insulin-like growth factor-1; MASH, metabolic dysfunction–associated steatohepatitis; MASLD, metabolic dysfunction–associated steatotic liver disease; RA, receptor agonist; RCT, randomised controlled trial; SGLT2, sodium-glucose cotransporter-2; T2DM, type 2 diabetes mellitus; THR-β, thyroid hormone receptor-β.

Only resmetirom and semaglutide currently hold regulatory approval for MASH. In both cases this is US FDA accelerated approval, restricted to noncirrhotic disease with moderate-to-advanced (F2–F3) fibrosis and contingent on confirmatory outcome data. All other agents are listed at their present stage of clinical development and should not be regarded as clinically actionable for MASLD.

↑, increased; ↓, decreased.

First, sustained weight reduction of ≥10%, achieved through lifestyle modification, remains the most broadly effective intervention and the backbone on which pharmacotherapy is layered, since it simultaneously reduces the substrate load, adipose stress, and insulin resistance that drive multiple axes (95).

Second, only two pharmacological agents currently hold regulatory approval for MASH, both granted accelerated approval by the US FDA for noncirrhotic disease with moderate-to-advanced (F2–F3) fibrosis and both contingent on confirmatory outcome data: the GLP-1 receptor agonist semaglutide and the liver-selective thyroid hormone receptor-β agonist resmetirom. Their approval supports the principle that correcting axis-specific endocrine dysfunction can produce measurable hepatic benefit, though both approvals are accelerated and await confirmatory outcome data.

Third, the remaining agents span a spectrum of evidence and must not be conflated with approved therapy. The dual incretin agonist tirzepatide has completed phase 2 histological evaluation with encouraging MASH-resolution and fibrosis outcomes and is now in phase 3 outcome trials; FGF21 analogues (efruxifermin, pegozafermin) have advanced through phase 2b to phase 3, with encouraging hepatic and fibrosis outcomes; non-steroidal FXR agonists and engineered FGF19 analogues (aldafermin) and tesamorelin/GH remain investigational; and agents targeting glucocorticoid amplification (11β-HSD1 inhibitors), cellular senescence (senolytics), and adipose thermogenesis (β3-adrenergic agonists) remain largely experimental, with mixed or preliminary data. **Table 1** summarises these agents by class, mechanism, target axis, and evidence stage.

Finally, because MASLD arises from several endocrine axes acting in concert, the most important therapeutic direction is likely to be combinatorial and phenotype-guided: matching agents to the dominant endocrine drivers in a given patient and combining mechanisms that act on distinct axes.

The relative contribution of each axis across the histological stages of disease, and the stages at which the currently approved agents act, is summarised in **Figure 2**.

**Figure 2:**
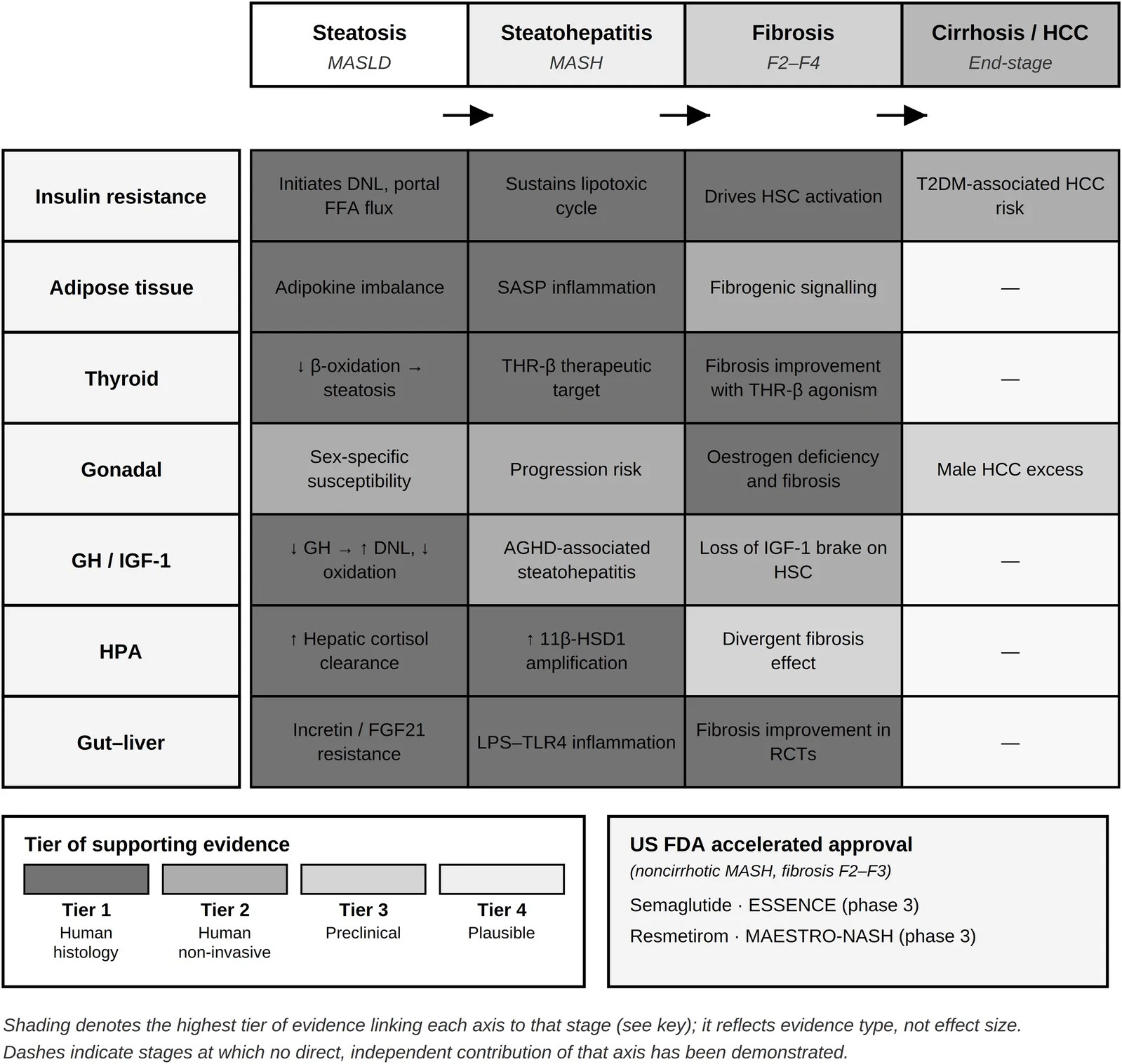
Contribution of the individual endocrine axes across the histological stages of MASLD.

Each row represents an endocrine axis, and each column a stage of disease progression, from simple steatosis through steatohepatitis (MASH) and fibrosis to cirrhosis and hepatocellular carcinoma (HCC). Shading denotes the highest tier of evidence linking each endocrine axis to the corresponding histological stage, on a four-level scale. Tier 1 (darkest): the association is supported by human histological or biopsy-defined outcomes, including interventional trials with liver-histology endpoints. Tier 2: supported by human observational data using validated non-invasive measures (imaging-based steatosis or fibrosis assessment, or serum biomarkers) but without histological confirmation. Tier 3: supported predominantly by preclinical mechanistic data with limited or indirect human corroboration. Tier 4 (palest): mechanistically plausible but not directly established at that stage. Dashes indicate stages at which no direct, independent contribution of that axis has been demonstrated. The scale reflects the nature of the available evidence, not effect size, and should not be read as a quantitative ranking of importance. Insulin resistance and adipose tissue dysfunction operate across the whole spectrum, whereas the HPA axis shows a stage-dependent pattern, with enhanced hepatic cortisol clearance in simple steatosis giving way to local 11β-hydroxysteroid dehydrogenase type 1 (11β-HSD1)-mediated glucocorticoid amplification in steatohepatitis, and the GH/IGF-1 axis contributes to steatosis through loss of direct GH action on hepatocytes and to fibrogenesis through loss of the IGF-1 brake on stellate cell activation. The panel at the lower right shows the two agents with US FDA accelerated approval for noncirrhotic MASH with F2-F3 fibrosis, with their pivotal trials: semaglutide and resmetirom.

## 6 Limitations

This review has limitations inherent to its narrative design. The synthesis was based on a purposive search of PubMed, Europe PMC, and Google Scholar for English-language literature on the endocrine determinants of MASLD, prioritising systematic reviews, meta-analyses, randomised controlled trials, and major society guidelines; it did not follow a formal systematic-review protocol, so selection bias and incomplete capture of relevant studies cannot be excluded. The evidence base is also heterogeneous across the endocrine axes, limiting direct comparison and precluding quantitative synthesis. Several of the mechanisms discussed, particularly those involving adipose browning, circadian glucocorticoid signalling, fibroblast growth factor and bile acid pathways, and the gut microbiota, rest substantially on preclinical or early-phase data, and their translation to routine clinical practice remains incomplete.

## 7 Conclusion

MASLD is best understood as a disease in which liver-intrinsic pathology is substantially governed by integrated endocrine-metabolic dysregulation. Insulin resistance remains the central engine, but its trajectory is shaped by adipose senescence and adipokine imbalance, reduced thyroid hormone sensitivity, gonadal steroid dimorphism, pre-receptor glucocorticoid amplification, and disrupted gut–liver signalling, axes that converge on shared intrahepatic processes of lipotoxicity, mitochondrial dysfunction, inflammasome activation, and impaired lipophagy, which drive steatosis towards steatohepatitis and fibrosis. Biological sex, early-life programming, and ageing further modify these determinants across the life-course, together accounting for much of the disease’s clinical heterogeneity.

Recognising this multi-hormonal structure changes how MASLD is screened, classified, and managed. It supports bidirectional case-finding, using a MASLD diagnosis to prompt endocrine evaluation and endocrine disorders to trigger assessment of hepatic fibrosis. It also shifts risk stratification beyond BMI towards visceral adiposity, hormone-sensitivity markers, and body composition. Therapeutically, the field has entered a transformative era in which sustained weight loss is complemented by a first generation of endocrine-targeted agents acting on distinct axes, raising the realistic prospect of MASH resolution and fibrosis regression. The central priorities now are to validate biomarkers that map endocrine phenotype to optimal therapy, to resolve tissue-specific hormone signalling, and to design phenotype-targeted, sex- and life-stage-aware trials. An endocrine framework offers not only a coherent account of MASLD pathogenesis but also a rational foundation for precision therapeutics.
